# Bioinformatics Analysis of the Effects of Tobacco Smoke on Gene Expression

**DOI:** 10.1371/journal.pone.0143377

**Published:** 2015-12-02

**Authors:** Chunhua Cao, Jianhua Chen, Chengqi Lyu, Jia Yu, Wei Zhao, Yi Wang, Derong Zou

**Affiliations:** 1 Department of Stomatology, Shanghai Jiao Tong University Affiliated Sixth People’s Hospital, Shanghai, 200233, China; 2 Shanghai Key Laboratory of Psychotic Disorders, Shanghai Mental Health Center, Shanghai Jiao Tong University School of Medicine, Shanghai, 200030, China; Wageningen UR Livestock Research, NETHERLANDS

## Abstract

This study was designed to explore the effects of tobacco smoke on gene expression through bioinformatics analyses. Gene expression profile GSE17913 was downloaded from the Gene Expression Omnibus database. The differentially expressed genes (DEGs) in buccal mucosa tissues between 39 active smokers and 40 never smokers were identified. Gene Ontology (GO) and pathway enrichment analyses of DEGs were performed, followed by protein-protein interaction (PPI) network, transcriptional regulatory network as well as miRNA-target regulatory network construction. In total, 88 up-regulated DEGs and 106 down-regulated DEGs were identified. Among these DEGs, cytochrome P450, family 1, subfamily A, polypeptide 1 (*CYP1A1*) and *CYP1B1* were enriched in the Metabolism of xenobiotics by cytochrome P450 pathway. In the PPI network, tyrosine 3-monooxygenase/tryptophan 5-monooxygenase activation protein, zeta (*YWHAZ*), and *CYP1A1* were hub genes. In the transcriptional regulatory network, transcription factors of MYC associated factor X (MAX) and upstream transcription factor 1 (USF1) regulated many overlapped DEGs. In addition, protein tyrosine phosphatase, receptor type, D (*PTPRD*) was regulated by multiple miRNAs in the miRNA-DEG regulatory network. *CYP1A1*, *CYP1B1*, *YWHAZ* and *PTPRD*, and TF of MAX and USF1 may have the potential to be used as biomarkers and therapeutic targets in tobacco smoke-related pathological changes.

## Introduction

There are approximately 1.3 billion people who smoke cigarettes in the world. Tobacco is an important risk factor for multiple human malignancies, which causes almost 5 million preventable deaths every year [1, 2]. More than 100 tumor promoters and carcinogens have been identified in tobacco [3]. Tobacco combustion products contain polycyclic aromatic hydrocarbons (PAHs), which have been suggested to be carcinogenic [4]. A variety of cancers such as lung cancer, oral cavity cancer, esophageal cancer and liver cancer are attributed to cigarette smoke [5]. In addition, smoking can reduce the efficacy of targeted anticancer therapies by stimulating metabolic clearance [6, 7]. A better understanding of the mechanisms underlying these diseases may lead to more effective treatments for cancer patients.

Previous studies have suggested that tobacco smoke causes a field of injury in the oral mucosa epithelial cells, which are among the most important physiological barriers against several exogenous stimuli [8]. Tobacco smoke has been found to affect gene expression in many tissues and cells, including epithelial cells [9]. Some studies have reported that histologically normal large airway epithelial cells of smokers display allelic loss [10] and p53 mutations [11]. An in vitro study by Pickett et al. [12] found that when a single source of human airway epithelial cells was exposed to the same dose of cigarette smoke condensate from 10 different cigarettes, 21 genes were altered by 9 of the 10 cigarettes. In addition, based on the transcriptome profiling, Spira et al. [13] have indicated that smoking induces the expression of genes involved in redox stress and xenobiotic metabolism in the large airway epithelial cells. Theoretically, the development of a transcriptome-based biomarker to identify high-risk smokers may provide a basis to protect against the carcinogenic effects of cigarette smoke. However, related studies are far from being enough.

In the current study, we downloaded the microarray data GSE17913 and identified the differentially expressed genes (DEGs) in buccal mucosa tissues between 39 active smokers and 40 never smokers. We performed pathway enrichment analysis and protein-protein interaction (PPI) network construction. The transcriptional regulatory network and the miRNA-target regulatory network were constructed. We aimed to define the effects of smoking on the oral epithelial cells. The findings of this study may provide new insights into the carcinogenic effects of smoking and then develop useful preventive strategies.

## Data and Methods

### Affymetrix microarray data

The microarray data GSE17913 [14] was downloaded from the Gene Expression Omnibus (GEO, http://www.ncbi.nlm.nih.gov/geo/) database based on the platform of the Affymetrix Human Genome U133 Plus 2.0 Array (Affymetrix Inc., Santa Clara, California, USA). The dataset consists of 79 samples, including 40 samples of buccal mucosa from never smokers (< 100 cigarettes per lifetime; 20 never smoker females; 20 never smoker males) and 39 samples of buccal mucosa from active smokers (≥ 15 pack year exposure; 19 smoker females; 20 smoker males). The demographic characteristics of never smokers and smokers were shown in Table 1.

**Table 1:** Demographic characteristics of never smokers and smokers.

	Female (N = 20)	Male (N = 20)	P value
**Never smokers**			
**Age (years) median**	45	45	0.51
**Age (years) range**	26–66	30–55	
**Smokers**			
**Age (years) median**	43	45.5	0.87
**Age (years) range**	27–63	30–54	
**Pack-year median**	25	32.5	0.37
**Pack-year range**	15–66	15–60	

### Data preprocessing and differential expression analysis

The original array data were preprocessed with background correction and quartile data normalization by robust multiarray average (RMA) [15]. Then, the probes were mapped to the gene symbols based on the microarray annotation information in R Bioconductor package hgu133a2.db, and the expression values of multiple probes for a given gene were reduced to a single value by taking their median expression value.

The paired t-test based on the limma package [16] in Bioconductor was used to identify the DEGs between active smokers and never smokers. The Benjamini & Hochberg method was used to decrease the false positive rate of the p-value, and the false discovery rate (FDR) was calculated. FDR < 0.05 was considered the cutoff value.

### Gene ontology and pathway enrichment analyses

The clusterProfiler (available at http://bioconductor.org/packages/release/bioc/html/clusterProfiler.html) [17] package in R is used to automate the process of biological term classification and the enrichment analysis of gene clusters. To analyze the DEGs at the functional level, Gene Ontology Biological Process (GO BP) and Kyoto Encyclopedia of Genes and Genomes (KEGG) pathway enrichment analyses were performed using the clusterProfiler tool. P-value < 0.05 was set as the threshold value. In addition, the pathway figure was described using pathview (available at http://bioconductor.org/packages/release/bioc/html/pathview.html and at http://Pathview.r-forge.r-project.org/) [18] in the R Bioconductor package.

### PPI network construction

The Search Tool for the Retrieval of Interacting Genes (STRING) database (http://string-db.org/) [19] is a precomputed global resource used to evaluate PPI information. In this paper, the STRING online tool was applied to analyze the PPI of DEGs, and only experimentally validated interactions with a combined score > 0.4 were selected as significant. The PPI network was constructed using cytoscape [20].

### Transcriptional regulatory network construction

The ENCyclopedia Of DNA Elements (ENCODE, http://genome.ucsc.edu/ENCODE/) Project aimed to provide comprehensive annotations of candidate functional elements in the human genome for scientific and medical communities [21]. In this study, we extracted all the human transcription factor (TF) binding sites were extracted from the ENCODE database. Next, the repeatability of each TF binding site was analyzed, and the TF binding site that appeared in at least 2 independent samples was selected for further analysis. Then, combining with the annotation data of genetic transcription area, we screened out all the TFs located in the gene promoter regions (1 kb upstream of the transcription start site (TSS) to 0.5 kb downstream of TSS). The TF-gene pairs were then constructed. Finally, we extracted the TF-DEG pairs from the TF-gene pairs and constructed the DEG-associated transcriptional regulatory network.

### miRNA-target regulatory network construction

The starBase v2.0 (http://starbase.sysu.edu.cn/) database provides the RNA-RNA and protein-RNA interaction networks from 108 CLIP-Seq (PAR-CLIP, HITS-CLIP, iCLIP, CLASH) data sets generated by 37 independent studies [22]. It also contains the miRNA-target regulatory networks which are verified by experiment and predicted by 5 algorithms (TargetScan, miRanda, Pictar2, PITA and RNA22). In our study, we screened out the miRNA-target pairs that were not only verified by at least 1 experiment but also predicted by at least 3 algorithms. Then the miRNA-target regulatory network was constructed.

## Results

### Identification of DEGs

**Fig 1:**
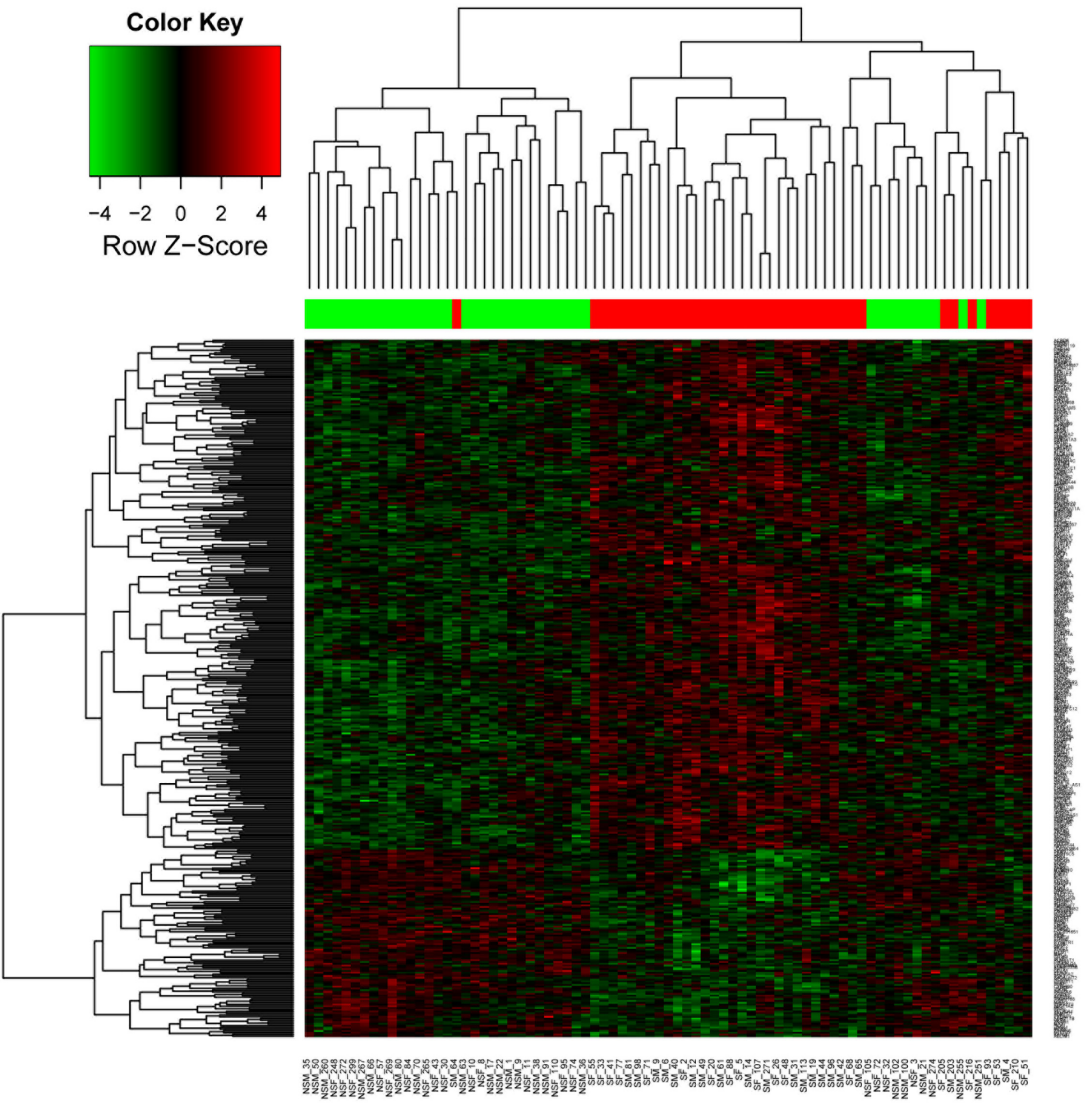
Heatmap plot of differentially expressed genes across all samples. Upper color bar represents sample classes; red represents smoker group; green represents non-smoker group.

A total of 394 DEGs were identified between active smokers and never smokers, of which 288 were up-regulated and 106 were down-regulated (S1 Table). The results are shown in the heatmap (Fig 1).

### GO and pathway enrichment analyses of DEGs

**Fig 2:**
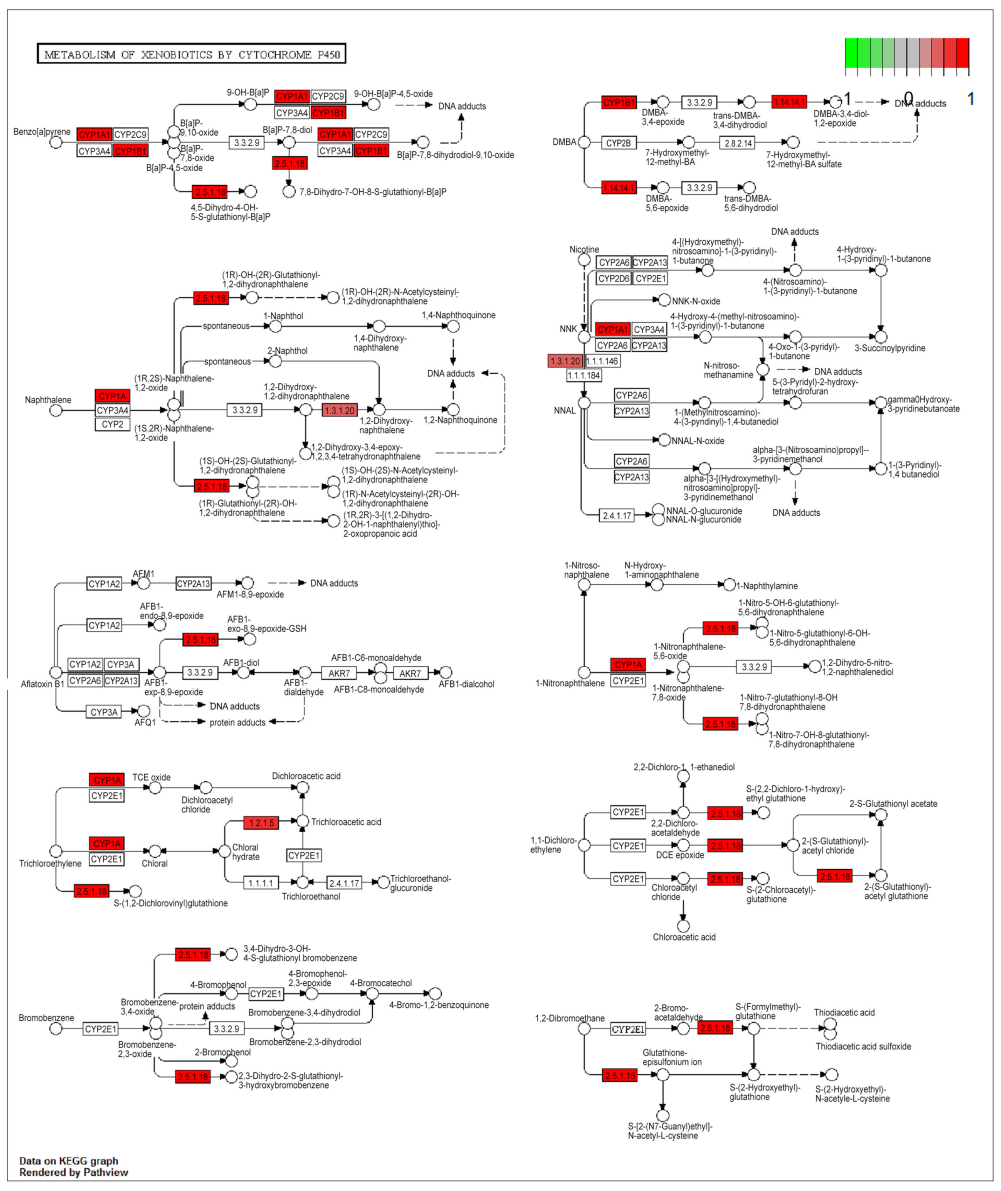
Differentially expressed gene (DEG) distribution in metabolism of xenobiotics by cytochrome P450 pathway. Red represents DEGs, and color shade represents log FC variation.

After pathway enrichment analysis, 3 pathways were obtained, namely the Metabolism of xenobiotics by cytochrome P450, Osteoclast differentiation, and Chemokine signaling pathway (Table 2). The Metabolism of xenobiotics by cytochrome P450 pathway was enriched by several DEGs including cytochrome P450, family 1, subfamily A, polypeptide 1 (*CYP1A1*) and *CYP1B1*. Specifically, the DEG distribution in the pathway of Metabolism of xenobiotics by cytochrome P45 was shown in Fig 2. Additionally, the DEGs were mainly enriched in GO BP terms related to biological processes, cellular processes and single-organism processes.

**Table 2:** Pathway enrichment result for differentially expressed genes (DEGs).

ID	Description	P-value	Gene Symbol
hsa00980	Metabolism of xenobiotics by cytochrome P450	2.80E-05	CYP1B1/CYP1A1/ALDH3A1/GSTA3/AKR1C2/AKR1C4/GSTM4/AKR1C3/GSTM3
hsa04380	Osteoclast differentiation	2.61E-02	NCF4/PPARG/SOCS3/TNFRSF11A/BLNK/AKT3/TYK2
hsa04062	Chemokine signaling pathway	2.76E-02	CCL5/CCL26/CCL18/CCR2/AKT3/JAK2/CCR7/CCL22/JAK3

All GO BP and KEGG pathways for the DEGs were shown in S2 Table.

### PPI network construction

**Fig 3:**
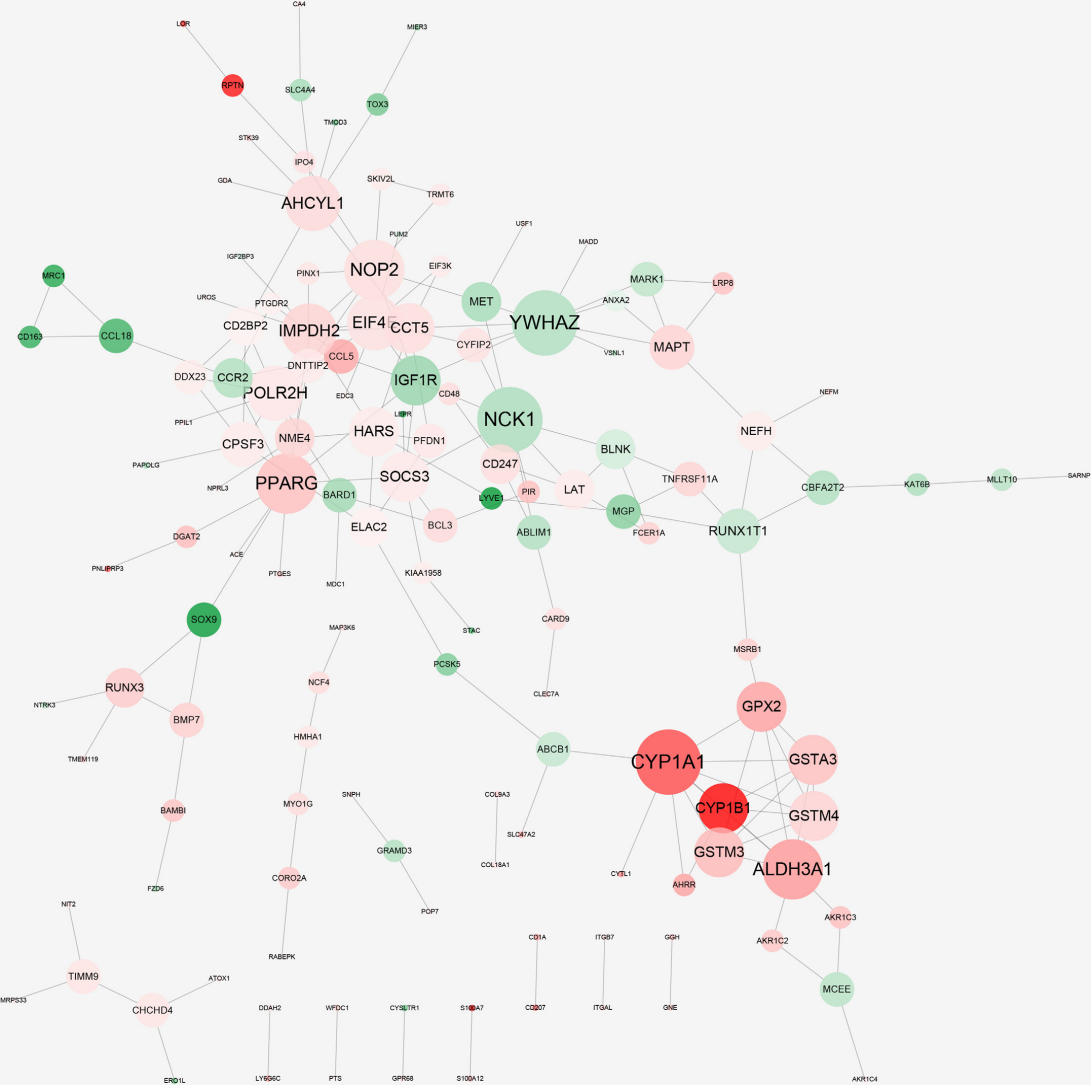
Protein-protein interaction (PPI) network constructed by the differentially expressed genes (DEGs). Node size represents node degree; a larger size indicates a larger degree. Red represents up-regulation, and green represents down-regulation.

The constructed PPI network was shown in Fig 3. The PPI network consisted of 139 nodes and 183 edges (S3 Table). Among these gene nodes, tyrosine 3-monooxygenase/tryptophan 5-monooxygenase activation protein, zeta (*YWHAZ*), NCK adaptor protein 1 (*NCK1*) and cytochrome P450, family 1, subfamily A, polypeptide 1 (*CYP1A1*) had the highest degrees of 9. Moreover, *YWHAZ* and *NCK1* were slightly down-regulated, and *CYP1A1* was significantly up-regulated.

### Transcriptional regulatory network of DEGs

**Fig 4:**
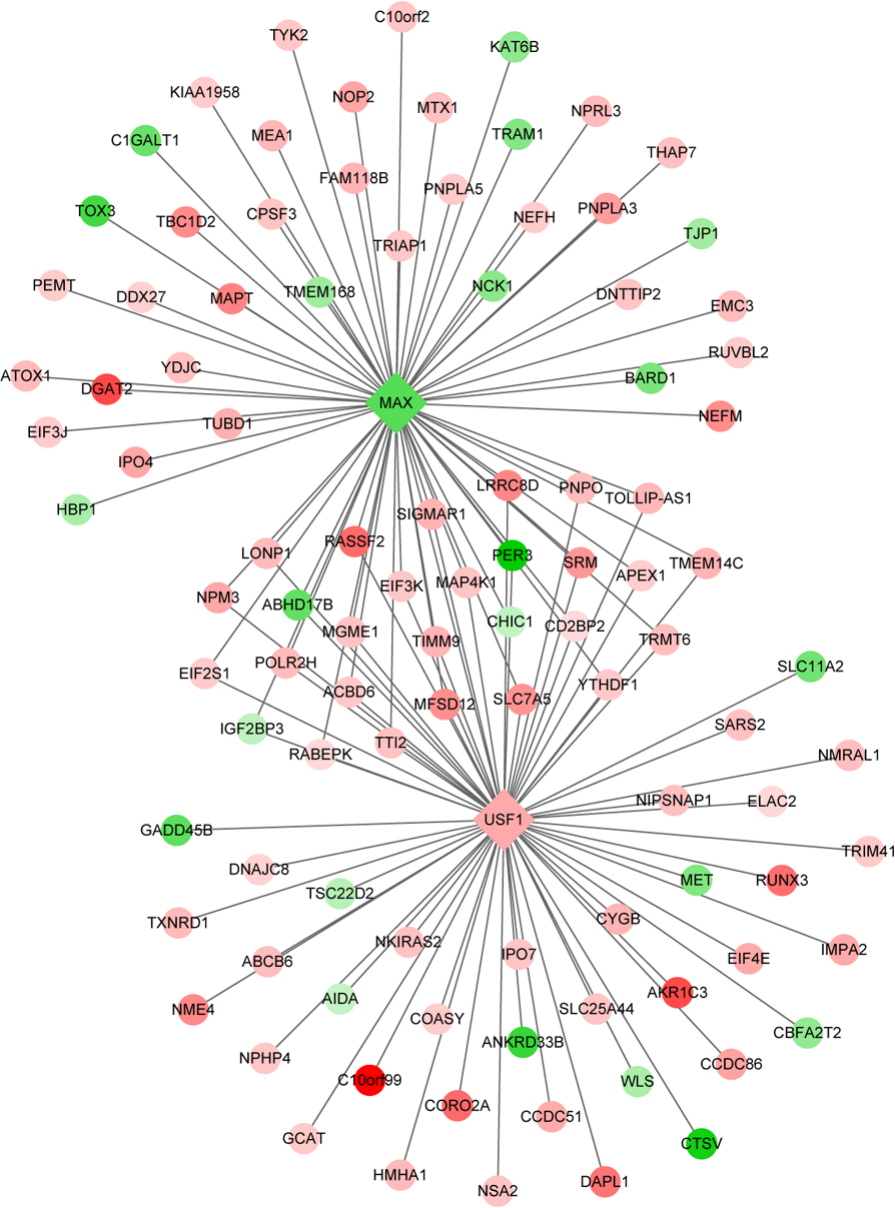
Transcriptional regulatory network for transcription factors of MAX and USF1. Diamond represents transcription factor, and circle represents differentially expressed genes (DEGs). Red represents up-regulated expression, and green represents down-regulated expression.

The DEG-associated transcriptional regulatory network was shown in Fig 4. The network consisted of 2 TFs, 101 target DEGs and 131 edges (S4 Table). Fig 4 showed that the TF MYC associated factor X (MAX) was down-regulated and upstream transcription factor 1 (USF1) was up-regulated, with many overlapped DEGs regulated by the 2 TFs.

### miRNA-DEG regulatory network

In total, 210 miRNAs, 2 TFs and 118 genes were included in the miRNA-DEG regulatory network (S5 Table). Almost every miRNA regulated two DEGs, but several DEGs were also regulated by multiple miRNAs, such as mesoderm induction early response 1, family member 3 (*MIER3*), cysteine-rich hydrophobic domain 1 (*CHIC1*) and protein tyrosine phosphatase, receptor type, D (*PTPRD*).

## Discussion

The identification of DEGs between smokers and never smokers is important to understanding the mechanisms underlying the carcinogenic effects of smoking. In this study, 288 up-regulated DEGs and 106 down-regulated DEGs were identified. Among these DEGs, *CYP1A1* and *CYP1B1* were mainly enriched in the pathway of Metabolism of xenobiotics by cytochrome P450. In the PPI network, *YWHAZ*, *NCK1* and *CYP1A1* were hub genes. In the transcriptional regulatory network, the TFs MAX and USF1 regulated many overlapping DEGs. In addition, *PTPRD* was regulated by multiple miRNAs in the miRNA-DEG regulatory network.

Cytochrome P450 enzymes can catalyze the biotransformation of various xenobiotic compounds to form ultimate toxicants [23]. Mutations in certain *CYP* genes contribute to clinically relevant diseases including malignancy [24]. In this study, Metabolism of xenobiotics by cytochrome P450 was a significant pathway and was enriched by several DEGs, including *CYP1B1* and *CYP1A1*. As mentioned above, the combustion products of tobacco contain PAHs, which can be converted into reactive metabolites via *CYP1A1*. These reactive metabolites may be involved in the initiation of carcinogenesis *via* the formation of bulky PAH-DNA adducts [25, 26]. In the human lung, high expression of *CYP1A1* has been associated with increased lung cancer risk [27]. Additionally, *CYP1B1* can also activate various carcinogens: for instance, *CYP1B1* can catalyze the formation of dihydrodiols of specific PAHs and their subsequent oxidation into carcinogenic dihydrodiol epoxides [28]. *CYP1B1* is also commonly overexpressed in human malignancies [29]. Our result was consistent with the findings above, and therefore, *CYP1A1* and *CYP1B1* may be potential targets in smoking-mediated malignancies.

In the PPI network, *YWHAZ* was one of the hub genes with the highest degree. Its encoded proteins are involved in many vital cellular processes such as signal transduction, metabolism, cell cycle regulation and apoptosis. *YWHAZ* protein expression is well known to be related to advanced disease grade and poor clinical outcome in lung cancer patients [30]. Research has found that YWHAZ is a potential regulator of the function of β-catenin, which is a central effector of Wnt signaling in tumorigenesis and metastasis [31]. In particular, tobacco smoke exposure may lead to the translocation of β-catenin via cooperation with interleukin-1β [32]. Taken together, *YWHAZ* may be a marker gene in tobacco smoke-related pathological changes.

Furthermore, in the transcriptional regulatory network, the TFs f MAX and USF1 regulated many overlapped DEGs. MAX is a member of the basic helix-loop-helix leucine zipper (bHLHZ) family of transcription factors. It can form heterodimers with other family members, including MYC, which is an oncoprotein implicated in cell growth, proliferation, differentiation and apoptosis [33]. The dysregulated expression of MYC has been reported in a wide range of human malignancies [34]. On the other hand, USF1 also encodes a member of the bHLHZ family and functions as a cellular transcription factor that regulates the expression of numerous genes involved in cellular proliferation and the cell cycle [35, 36]. Importantly, Wu et al. [37] have demonstrated that nicotine, a component of tobacco smoke, can enhance USF1 translocation from the cytoplasm to the nucleus. As a result, we speculated that tobacco smoke might induce cancer by targeting genes such as MAX and USF1.

In the miRNA-DEG regulatory network, several DEGs were regulated by multiple miRNAs, such as *PTPRD*. The protein encoded by *PTPRD* is a member of the protein tyrosine phosphatase (PTP) family, signaling molecules that regulate cell growth, differentiation, and oncogenic transformation [38]. *PTPRD* mutations have been found in lung cancer and other malignancies [39]. Currently, there are few reports about the relationship between tobacco smoke and the dysregulated expression of *PTPRD*. Thus, we speculated that *PTPRD* might be a potential biomarker associated with the carcinogenic effect of tobacco smoke.

In conclusion, we have used a bioinformatics approach to identify the marker genes related to the carcinogenic role of tobacco smoke. These DEGs, such as *CYP1A1*, *CYP1B1*, *YWHAZ* and *PTPRD*, and TF of MAX and USF1, may have the potential to be used as biomarkers and therapeutic targets in tobacco smoke-related pathological changes. The findings in this study may contribute to our further understanding of the underlying molecular mechanisms that are modulated by tobacco smoke. Further genetic and experimental studies with larger sample sizes are still needed to confirm the results.

## Supporting Information

S1 Table All identified differentially expressed genes (DEGs) between active smokers and never smokers. (XLS)

S2 Table All Gene Ontology Biological Processes (GO BP) and Kyoto Encyclopedia of Genes and Genomes (KEGG) pathways for the DEGs. (XLS)

S3 Table All the nodes and edges in the protein-protein interaction (PPI) network. (XLS)

S4 Table All the nodes and edges in the transcriptional regulatory network. (XLS)

S5 Table All the nodes and edges in the microRNA regulatory network. (XLS)
