# Correction: Bioinformatics Analysis of the Effects of Tobacco Smoke on Gene Expression

**DOI:** 10.1371/journal.pone.0150778

**Published:** 2016-03-02

**Authors:** Chunhua Cao, Jianhua Chen, Chengqi Lyu, Jia Yu, Wei Zhao, Yi Wang, Derong Zou

There is an error in the fifth sentence of the Abstract. The correct sentence is: In total, 288 up-regulated DEGs and 106 down-regulated DEGs were identified.

There is an error in the third sentence of the Results section under the heading “GO and pathway enrichment analyses of DEGs.” The correct sentence is: Specifically, the DEG distribution in the pathway of Metabolism of xenobiotics by cytochrome P450 was shown in Fig 2.
